# Supplementary material for: Bidirectional Associations between Parental Feeding Practices and Child Eating Behaviors in a Chinese Sample
Source: Nutrients. 2023 Dec 22;16(1):44. doi: 10.3390/nu16010044 (PMC10780825; doi:10.3390/nu16010044)
Supplement: Supplementary file 1 [file nutrients-16-00044-s001.zip › nutrients-2763008-supplementary.pdf]

Supplementary materials

Supplementary Table S1. Bivariate analysis with caregivers and child characteristics, as independent variables, and caregivers' feeding practices, child eating behaviors, as the dependent variable.

**Variables at T1**	**Encouragement of Healthy Eating**	**Modelling**	**Monitoring**	**Content Restricted Feeding**	**Pressure to Eat**	**Food as a Reward**	**Food Fussiness**	**Food Responsiveness**	**Satiety Responsiveness**	**Unhealthy Eating Habits**	**Initiative Eating**
**Child sex**											
Boys	4.08±0.51	4.11±0.59	4.05±0.82	3.42±0.82	3.24±0.83	3.22±0.75	3.03±0.63	2.44±0.53	2.68±0.69	2.53±0.81	3.43±0.67
Girls	4.15±0.50	4.13±0.54	4.07±0.79	3.48±0.77	3.19±0.87	3.32±0.75	2.97±0.67	2.45±0.61	2.62±0.67	2.44±0.81	3.49±0.68
t	-1.898	-0.482	-0.427	-1.105	0.883	-1.785	1.348	-0.142	1.323	1.584	-1.143
P	0.058	0.630	0.669	0.269	0.377	0.075	0.178	0.887	0.186	0.114	0.253
**Child weight status**											
Underweight	4.40±0.31	4.20±0.71	4.25±1.04	3.61±0.87	3.21±0.71	3.14±0.90	2.58±0.66	2.31±0.45	2.82±0.72	2.54±0.85	3.54±0.64
Normal weight	4.12±0.50	4.12±0.55	4.06±0.77	3.45±0.78	3.26±0.83	3.28±0.74	3.00±0.64	2.41±0.54	2.69±0.67	2.55±0.81	3.41±0.67
Overweight or Obesity	4.04±0.59	4.08±0.60	3.98±0.89	3.42±0.87	3.00±0.94	3.27±0.80	3.12±0.65	2.64±0.68	2.39±0.67	2.21±0.73	3.69±0.66
F	5.570	0.560	1.420	0.571	5.257	0.390	7.573	10.368	12.937	10.214	10.184
P	0.004**	0.571	0.242	0.565	0.005**	0.677	0.001**	0.000***	0.000***	0.000***	0.000***
**The role of caregivers**											
Mothers	4.12±0.51	4.10±0.58	4.05±0.81	3.45±0.80	3.19±0.86	3.26±0.76	3.01±0.64	2.43±0.56	2.65±0.69	2.50±0.82	3.45±0.67
Fathers	4.07±0.50	4.18±0.49	4.08±0.78	3.44±0.78	3.36±0.79	3.32±0.70	2.98±0.70	2.53±0.61	2.63±0.67	2.44±0.76	3.51±0.66

Variables at T1	Encouragement of Healthy Eating	Modelling	Monitoring	Content Restricted Feeding	Pressure to Eat	Food as a Reward	Food Fussiness	Food Responsiveness	Satiety Responsiveness	Unhealthy Eating Habits	Initiative Eating
t	1.119	-1.638	-0.432	0.190	-2.380	-1.033	0.598	-2.007	0.240	0.780	-1.083
P	0.263	0.102	0.666	0.849	0.018*	0.302	0.550	0.045*	0.811	0.435	0.279
**Duration of breastfeeding**											
0-6 months	4.07±0.54	4.07±0.59	4.06±0.77	3.46±0.80	3.25±0.90	3.39±0.77	3.00±0.67	2.44±0.62	2.62±0.75	2.53±0.85	3.47±0.68
6-12 months	4.09±0.50	4.11±0.56	4.06±0.79	3.44±0.80	3.23±0.83	3.21±0.74	3.01±0.67	2.45±0.56	2.69±0.67	2.52±0.81	3.43±0.66
More than 12 months	4.20±0.48	4.18±0.54	4.04±0.87	3.47±0.81	3.15±0.82	3.24±0.73	2.99±0.65	2.44±0.53	2.58±0.61	2.38±0.75	3.51±0.68
F	4.436	2.006	0.041	0.125	0.829	4.217	0.050	0.011	2.178	2.233	0.864
P	0.012*	0.135	0.960	0.882	0.437	0.015*	0.951	0.989	0.114	0.108	0.422
**Caregivers' weight status**											
Underweight	4.06±0.49	4.01±0.57	4.00±0.90	3.35±0.94	3.14±0.89	3.19±0.71	3.02±0.60	2.43±0.42	2.62±0.70	2.53±0.76	3.45±0.62
Normal weight	4.12±0.49	4.12±0.56	4.06±0.78	3.42±0.81	3.24±0.84	3.26±0.76	3.00±0.66	2.42±0.57	2.65±0.67	2.50±0.82	3.46±0.68
Overweight or Obesity	4.10±0.56	4.14±0.59	4.06±0.84	3.57±0.73	3.18±0.86	3.32±0.74	3.01±0.65	2.51±0.59	2.64±0.72	2.45±0.81	3.49±0.67
F	0.490	1.320	0.195	3.678	0.685	1.019	0.028	1.745	0.060	0.407	0.151
P	0.613	0.268	0.823	0.026*	0.505	0.362	0.973	0.175	0.942	0.666	0.860
**Caregivers' education level**											

**Variables at T1**	**Encouragement of Healthy Eating**	**Modelling**	**Monitoring**	**Content Restricted Feeding**	**Pressure to Eat**	**Food as a Reward**	**Food Fussiness**	**Food Responsiveness**	**Satiety Responsiveness**	**Unhealthy Eating Habits**	**Initiative Eating**
Senior high school or below	4.07±0.51	4.11±0.59	4.10±0.78	3.58±0.80	3.15±0.91	3.20±0.75	3.09±0.57	2.48±0.56	2.78±0.69	2.56±0.87	3.44±0.74
College or higher	4.11±0.51	4.12±0.57	4.05±0.81	3.43±0.80	3.22±0.84	3.28±0.75	3.0±0.65	2.44±0.57	2.63±0.68	2.48±0.80	3.47±0.67
t	-0.777	-0.017	0.453	1.528	-0.710	-0.811	1.236	0.533	1.867	0.849	-0.377
P	0.437	0.986	0.651	0.127	0.478	0.418	0.217	0.595	0.062	0.396	0.706
**Number of the children**											
One	4.12±0.51	4.08±0.58	4.07±0.82	3.44±0.80	3.18±0.85	3.30±0.77	3.03±0.66	2.44±0.57	2.63±0.69	2.52±0.83	3.43±0.68
Two or more	4.10±0.52	4.18±0.54	4.04±0.78	3.47±0.79	3.29±0.84	3.23±0.72	2.96±0.63	2.45±0.56	2.68±0.68	2.43±0.78	3.51±0.67
t	0.594	-2.506	0.601	-0.526	-1.841	1.319	1.720	-0.196	-1.151	1.587	-1.762
P	0.553	0.012*	0.548	0.599	0.066	0.188	0.086	0.845	0.250	0.113	0.078
**Family structure**											
Living with parents and grandparents	4.06±0.53	4.09±0.57	4.03±0.83	3.44±0.82	3.23±0.84	3.29±0.72	3.07±0.66	2.47±0.56	2.66±0.67	2.57±0.79	3.42±0.67
Living with parents	4.17±0.49	4.15±0.57	4.09±0.77	3.47±0.77	3.21±0.86	3.25±0.78	2.93±0.62	2.42±0.58	2.64±0.69	2.40±0.82	3.51±0.68
t	-2.934	-1.587	-1.091	-0.620	0.306	0.628	3.318	1.188	0.533	2.953	-1.967
P	0.003**	0.113	0.276	0.536	0.760	0.530	0.001**	0.235	0.594	0.003**	0.049*

Variables at T1	Encouragement of Healthy Eating	Modelling	Monitoring	Content Restricted Feeding	Pressure to Eat	Food as a Reward	Food Fussiness	Food Responsiveness	Satiety Responsiveness	Unhealthy Eating Habits	Initiative Eating
**Household income/year**											
Below average	4.13±0.50	4.11±0.56	4.06±0.83	3.44±0.82	3.20±0.89	3.36±0.76	2.97±0.67	2.51±0.54	2.66±0.69	2.53±0.80	3.51±0.65
Above average	4.09±0.52	4.11±0.57	4.06±0.78	3.46±0.78	3.24±0.82	3.21±0.74	3.03±0.63	2.40±0.58	2.64±0.68	2.47±0.82	3.43±0.69
t	0.995	0.143	-0.061	-0.274	-0.621	2.705	-1.283	2.767	0.380	1.049	1.672
P	0.320	0.886	0.952	0.784	0.535	0.007**	0.200	0.006**	0.704	0.294	0.095
**Child age**											
r	-0.023	-0.009	-0.030	-0.025	-0.016	0.072	0.018	-0.022	0.063	0.020	-0.014
P	0.511	0.792	0.389	0.471	0.642	0.036*	0.591	0.513	0.067	0.553	0.676
**Child BMI Z score**											
r	-0.111**	-0.055	-0.047	-0.002	-0.144***	-0.004	0.068*	0.204***	-0.237***	-0.180***	0.125***
P	0.001	0.109	0.167	0.943	0.000	0.917	0.048	0.000	0.000	0.000	0.000
**Caregivers' age**											
r	-0.015	-0.020	0.012	-0.061	0.045	0.015	-0.060	-0.056	-0.013	0.003	-0.006
P	0.668	0.566	0.733	0.073	0.186	0.664	0.081	0.103	0.710	0.932	0.857
**Concern about child overweight**											
r	-0.098	-0.074	0.017	0.093	-0.110	0.020	0.013	0.235	-0.228	-0.210	0.189

Variables at T1	Encouragement of Healthy Eating	Modelling	Monitoring	Content Restricted Feeding	Pressure to Eat	Food as a Reward	Food Fussiness	Food Responsiveness	Satiety Responsiveness	Unhealthy Eating Habits	Initiative Eating
P	0.004**	0.031*	0.628	0.007**	0.001**	0.563	0.707	0.000***	0.000***	0.000***	0.000***
**Concern about child underweight**											
r	-0.039	0.016	0.057	-0.006	0.235	-0.038	0.093	-0.122	0.427	0.337	-0.230
P	0.249	0.644	0.095	0.854	0.000***	0.265	0.006**	0.000***	0.000***	0.000***	0.000***
**Perception of child weight**											
r	-0.076	-.030	-.020	0.038	-0.196	-0.006	-0.058	0.259	-0.406	-0.310	0.244
P	0.026*	0.384	0.563	0.271	0.000*	0.862	0.088	0.000***	0.000***	0.000***	0.000***
**Caregivers depression**											
r	-0.117	-0.142	-0.026	0.007	0.048	-0.030	0.082	0.061	0.166	0.126	-0.091
P	0.001**	0.000***	0.440	0.842	0.162	0.375	0.016*	0.074	0.000***	0.000***	0.008**
**Inhibition control**											
r	0.213	0.204	0.095	0.056	-0.054	0.055	-0.167	-0.069	-0.184	-0.220	0.222
P	0.000***	0.000***	0.005**	0.101	0.114	0.110	0.000***	0.044*	0.000***	0.000***	0.000***
**Emotionality**											
r	-0.123	-0.109	0.003	0.001	0.165	0.092	0.301	0.147	0.195	0.235	-0.147
P	0.000***	0.001**	0.921	0.984	0.000***	0.007**	0.000***	0.000***	0.000***	0.000***	0.000***

Notes. *$P<0.05$, **$P<0.01$, ***$P<0.001$.

Supplementary Table S2. Correlations between parental feeding practices and child eating behaviors (*n* = 853).

	T1 FF	T1 FR	T1 SR	T1 UE	T1 IE	T2 FF	T2 FR	T2 SR	T2 UE	T2 IE
T1 Encouragement of Healthy Eating	-0.199**	-0.030	-0.117**	-0.104**	0.148**	-0.196**	0.012	-0.108**	-0.122**	0.140**
T1 Modelling	-0.108**	-0.019	-0.143**	-0.190**	0.146**	-0.133**	-0.029	-0.083*	-0.190**	0.123**
T1 Monitoring	-0.058	0.017	-0.028	0.011	0.006	-0.060	0.056	-0.011	-0.059	0.024
T1 Content Restricted Feeding	-0.053	0.077*	-0.043	-0.074*	0.076*	-0.104**	0.060	-0.064	-0.134**	0.080*
T1 Pressure to Eat	0.128**	0.057	0.279**	0.397**	-0.301**	0.037	-0.007	0.209**	0.316**	-0.224**
T1 Use of Food as a Reward	0.115**	0.147**	0.043	0.120**	-0.021	0.090**	0.046	0.026	0.103**	-0.043
T2 Encouragement of Healthy Eating	-0.177**	-0.008	-0.099**	-0.088**	0.148**	-0.257**	0.022	-0.113**	-0.146**	0.202**
T2 Modelling	-0.097**	0.002	-0.082*	-0.129**	0.102**	-0.165**	0.013	-0.117**	-0.227**	0.211**
T2 Monitoring	-0.046	0.006	-0.013	0.000	0.044	-0.087*	0.009	-0.010	-0.083*	0.109**
T2 Content Restricted Feeding	-0.093**	-0.011	-0.081*	-0.049	0.025	-0.100**	0.002	-0.055	-0.134**	0.100**
T2 Pressure to Eat	0.083*	0.037	0.214**	0.313**	-0.239**	0.069*	0.099**	0.204**	0.315**	-0.234**
T2 Use of Food as a Reward	0.157**	0.094**	0.101**	0.136**	-0.076*	0.133**	0.120**	0.117**	0.190**	-0.038

***Notes*.** FF: food fussiness; FR: food responsiveness; SR: satiety responsiveness; UE: unhealthy eating habits; IE: initiative eating; *$P<0.05$, **$P<0.01$.

Supplementary Table S3. Model fit of each cross-lag model (*n* = 853).

Feeding practices	Eating behaviors	χ^2	*df*	CFI	RMSEA	RMR
Encouragement of Healthy Eating	Food Fussiness	105.991	18	0.905	0.076	0.057
Modelling	Food Fussiness	82.838	18	0.917	0.065	0.046
Monitoring	Food Fussiness	53.976	18	0.952	0.048	0.033
Content Restricted Feeding	Food Fussiness	43.321	18	0.964	0.041	0.026
Pressure to Eat	Food Fussiness	122.600	18	0.883	0.080	0.059
Use of Food as a Reward	Food Fussiness	63.210	20	0.939	0.054	0.032
Encouragement of Healthy Eating	Food Responsiveness	92.769	21	0.902	0.063	0.047
Modelling	Food Responsiveness	64.325	21	0.929	0.049	0.036
Monitoring	Food Responsiveness	38.222	21	0.970	0.031	0.024
Content Restricted Feeding	Food Responsiveness	38.268	21	0.969	0.031	0.023
Pressure to Eat	Food Responsiveness	113.678	21	0.888	0.072	0.063
Use of Food as a Reward	Food Responsiveness	48.256	21	0.954	0.039	0.027
Encouragement of Healthy Eating	Satiety Responsiveness	106.019	21	0.922	0.069	0.050
Modelling	Satiety Responsiveness	78.532	21	0.941	0.057	0.041
Monitoring	Satiety Responsiveness	50.473	21	0.968	0.041	0.028
Content Restricted Feeding	Satiety Responsiveness	48.036	21	0.970	0.039	0.025
Pressure to Eat	Satiety Responsiveness	110.091	21	0.919	0.071	0.065
Use of Food as a Reward	Satiety Responsiveness	61.369	21	0.956	0.047	0.031
Encouragement of Healthy Eating	Unhealthy Eating Habits	106.557	24	0.926	0.064	0.048
Modelling	Unhealthy Eating Habits	74.269	24	0.952	0.050	0.038

Monitoring	Unhealthy Eating Habits	48.281	24	0.975	0.034	0.025
Content Restricted Feeding	Unhealthy Eating Habits	44.990	24	0.978	0.032	0.022
Pressure to Eat	Unhealthy Eating Habits	105.504	24	0.933	0.063	0.060
Use of Food as a Reward	Unhealthy Eating Habits	54.479	24	0.969	0.039	0.026
Encouragement of Healthy Eating	Initiative eating	95.036	24	0.922	0.059	0.046
Modelling	Initiative eating	65	24	0.949	0.045	0.035
Monitoring	Initiative eating	37.152	24	0.982	0.025	0.022
Content Restricted Feeding	Initiative eating	35.365	24	0.984	0.024	0.019
Pressure to Eat	Initiative eating	98.341	24	0.921	0.060	0.057
Use of Food as a Reward	Initiative eating	47.118	24	0.968	0.034	0.024

Notes. CFI: Comparative Fit Index; RMSEA: Root Mean Square Error of Approximation; RMR: Root Mean-square Residual.

Supplementary Table S4. Strengthening the Reporting of Observational studies in Epidemiology (STROBE) guidelines for observational studies.

	Item No.	Recommendation	Page No.	Relevant text from manuscript
**Title and abstract**	1	(*a*) Indicate the study's design with a commonly used term in the title or the abstract	p. 1	"longitudinal study…"
		(*b*) Provide in the abstract an informative and balanced summary of what was done and what was found	p. 1	See Abstract
**Introduction**				
Background/rationale	2	Explain the scientific background and rationale for the investigation being reported	p. 2-2	See Introduction
Objectives	3	State specific objectives, including any prespecified hypotheses	p. 2	See Introduction
**Methods**				
Study design	4	Present key elements of study design early in the paper	p. 3	See Methods > Study design and participants
Setting	5	Describe the setting, locations, and relevant dates, including periods of recruitment, exposure, follow-up, and data collection	p. 3	See Methods > Study design and participants
Participants	6	(*a*) *Cohort study*—Give the eligibility criteria, and the sources and methods of selection of participants. Describe methods of follow-up<br>*Case-control study*—Give the eligibility criteria, and the sources and methods of case ascertainment and control selection. Give the rationale for the choice of cases and controls	p. 3	See Methods > Study design and participants

		Cross-sectional study—Give the eligibility criteria, and the sources and methods of selection of participants		
		(*b*) *Cohort study*—For matched studies, give matching criteria and number of exposed and unexposed<br>*Case-control study*—For matched studies, give matching criteria and the number of controls per case	n/a	n/a
Variables	7	Clearly define all outcomes, exposures, predictors, potential confounders, and effect modifiers. Give diagnostic criteria, if applicable	P. 3-4	See Methods > Demographic and socioeconomic data, feeding, eating, coviates.
Data sources/ measurement	8*	For each variable of interest, give sources of data and details of methods of assessment (measurement). Describe comparability of assessment methods if there is more than one group	p. 3-5	See Methods > Demographic and socioeconomic data, feeding, eating, coviates.
Bias	9	Describe any efforts to address potential sources of bias	p. 6-8	See Methods > Demographic and socioeconomic data, coviates.
Study size	10	Explain how the study size was arrived at	p.3	See Methods > Study design and participants
Quantitative variables	11	Explain how quantitative variables were handled in the analyses. If applicable, describe which groupings were chosen and why	p. 3,5	See Methods > Study design and participants, statistical analysis

Statistical methods	12	(*a*) Describe all statistical methods, including those used to control for confounding	p. 5	See Methods > statistical analysis
		(*b*) Describe any methods used to examine subgroups and interactions	n/a	n/a
		(*c*) Explain how missing data were addressed	p. 3,5	See Methods > Study design and participants
		(*d*) *Cohort study*—If applicable, explain how loss to follow-up was addressed<br>*Case-control study*—If applicable, explain how matching of cases and controls was addressed<br>*Cross-sectional study*—If applicable, describe analytical methods taking account of sampling strategy	p. 3,5	See Methods > statistical analysis
		(*e*) Describe any sensitivity analyses	n/a	n/a
**Results**				
Participants	13*	(a) Report numbers of individuals at each stage of study—eg numbers potentially eligible, examined for eligibility, confirmed eligible, included in the study, completing follow-up, and analysed	p. 3	See Methods > Study design and participants
		(b) Give reasons for non-participation at each stage	p. 3	See Methods > Study design and participants
		(c) Consider use of a flow diagram	n/a	n/a

Descriptive data	14*	(a) Give characteristics of study participants (eg demographic, clinical, social) and information on exposures and potential confounders	p. 5-6	See Results > Demographic and socioeconomic characteristics of the participants at baseline and Table 1
		(b) Indicate number of participants with missing data for each variable of interest	n/a	n/a
		(c) *Cohort study*—Summarise follow-up time (eg, average and total amount)	p. 5-7	See Results > Study and participant characteristics; Stability and continuity of parental feeding practices and child eating behaviors; and Table 1,2
Outcome data	15*	*Cohort study*—Report numbers of outcome events or summary measures over time	p. 5-7	See Results > Stability and continuity of parental feeding practices and child eating behaviors
		Case-control study—Report numbers in each exposure category, or summary measures of exposure	n/a	n/a
		Cross-sectional study—Report numbers of outcome events or summary measures	n/a	n/a

Main results	16	(*a*) Give unadjusted estimates and, if applicable, confounder-adjusted estimates and their precision (eg, 95% confidence interval). Make clear which confounders were adjusted for and why they were included	p. 7-10	See Results > Bidirectional associations of parental feeding practices with child eating behaviors; figure 1,2; supplementary table 1,2.<br>(Confounders were listed and explained in Background > while controlling for potential covariates; Result > Some demographic variables... were added to each cross-lagged model as covariates.)
		(*b*) Report category boundaries when continuous variables were categorized	p.6	See Results > table 1, 2; and supplementary table 1,2.
		(*c*) If relevant, consider translating estimates of relative risk into absolute risk for a meaningful time period	n/a	n/a
Other analyses	17	Report other analyses done—eg analyses of subgroups and interactions, and sensitivity analyses	n/a	n/a
**Discussion**				

Key results	18	Summarise key results with reference to study objectives	p. 7-10	See Discussion > The bidirectional associations of parental feeding practices with child eating behaviors
Limitations	19	Discuss limitations of the study, taking into account sources of potential bias or imprecision. Discuss both direction and magnitude of any potential bias	p. 13	See Discussion > There are several limitations...
Interpretation	20	Give a cautious overall interpretation of results considering objectives, limitations, multiplicity of analyses, results from similar studies, and other relevant evidence	p. 11-13	See Discussion, Conclusion
Generalisability	21	Discuss the generalisability (external validity) of the study results	p. 13	See Discussion > There are several limitations...
**Other information**				
Funding	22	Give the source of funding and the role of the funders for the present study and, if applicable, for the original study on which the present article is based	p. 14	See Funding information

*Give information separately for cases and controls in case-control studies and, if applicable, for exposed and unexposed groups in cohort and cross-sectional studies.

Note: An Explanation and Elaboration article discusses each checklist item and gives methodological background and published examples of transparent reporting. The STROBE checklist is best used in conjunction with this article (freely available on the Web sites of PLoS Medicine at http://www.plosmedicine.org/, Annals of Internal Medicine at http://www.annals.org/, and Epidemiology at http://www.epidem.com/). Information on the STROBE Initiative is available at www.strobe-statement.org.
